# Supplementary material for: Stimulus edges induce orientation tuning in superior colliculus
Source: Nat Commun. 2023 Aug 8;14:4756. doi: 10.1038/s41467-023-40444-1 (PMC10409754; doi:10.1038/s41467-023-40444-1)
Supplement: Supplementary file 1 — Supplementary Information [file 41467_2023_40444_MOESM1_ESM.pdf]

Stimulus edges induce orientation tuning in superior colliculus

Inventory of Supporting Information

Supplementary Fig. 1. Orientation-selective responses at the edge of circular grating stimuli are robust across animals.

Supplementary Fig. 2. Calcium transients and tuning curves for ROIs of six different preferred orientations.

Supplementary Fig. 3. Orientation-selective responses at the edge of grating stimuli persist over broad spatiotemporal frequency ranges.

Supplementary Fig. 4. Lack of orientation clustering away from edge.

Supplementary Fig. 5. Cortex-intact preparation and C-fos experiment.

Supplementary Fig. 6. Orientation and direction selectivity of SC neurons towards full-field drifting gratings.

Supplementary Fig. 7. SC interneurons exhibit the same edge-induced orientation selectivity.

Supplementary Fig. 8. Response and tuning properties of SC neurons towards drifting grating stimuli without edge, with vertical edge, or with horizontal edge.

Supplementary Fig. 9. Mouse primary visual cortex lacks similar edge-induced effects.

Supplementary Table 1. Imaging parameters associated with experimental data.

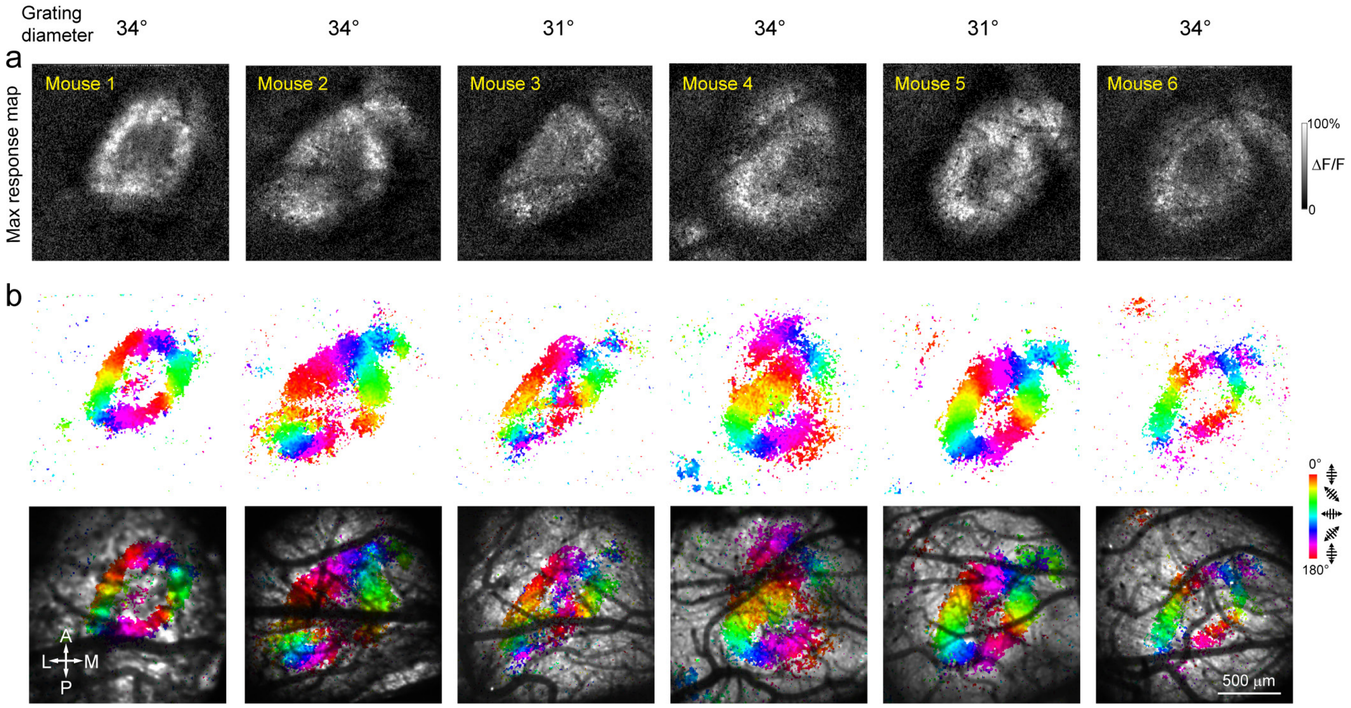

Supplementary Fig. 1. Orientation-selective responses at the edge of circular grating stimuli are robust across animals. (a) Maps of the maximal calcium response and (b) maps of orientation selective pixels (pixel size: 7 μm; color-coded by preferred orientation) evoked by circular gratings of similar sizes to Fig. 1 from the SC of 6 mice.

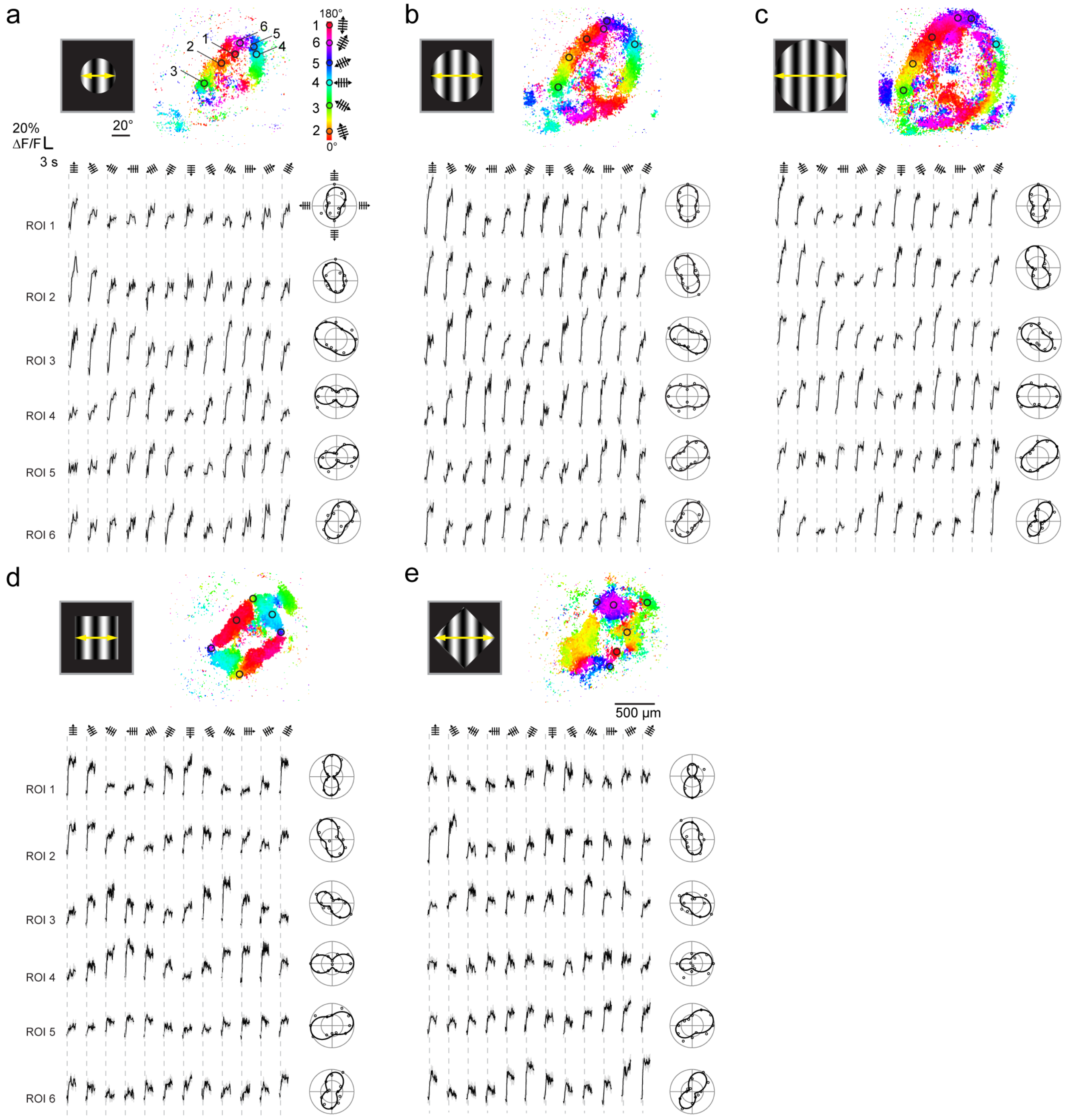

Supplementary Fig. 2. Calcium transients and tuning curves for ROIs of six different preferred orientations. (a-e) Drifting grating stimuli, OS map, and calcium transients (ΔF/F; ten trial average, black trace; s.e.m., gray shade) and their normalized polar plots for ROIs in Fig. 2.

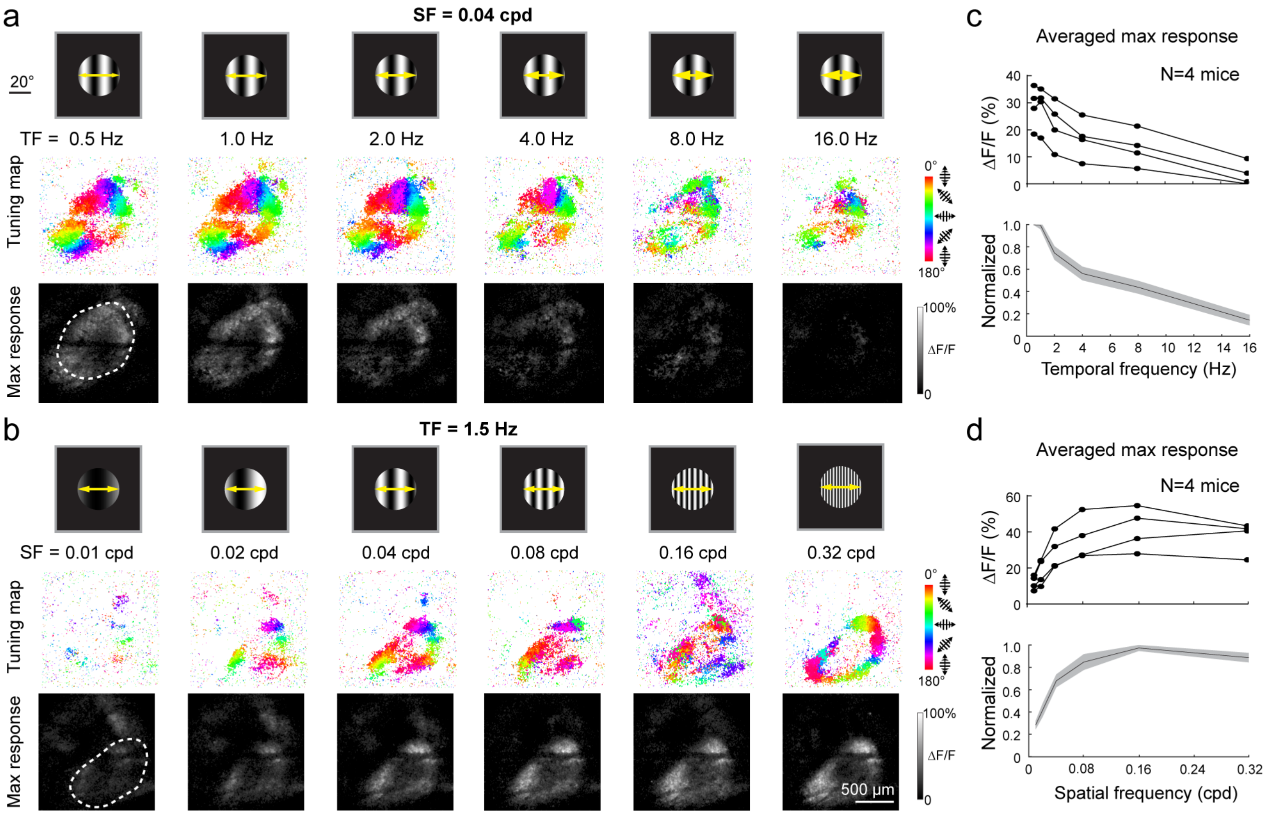

Supplementary Fig. 3 Orientation-selective responses at the edge of grating stimuli persist over broad spatiotemporal frequency ranges. (a, b) Circular gratings (a) of spatial frequency at 0.04 cpd and temporal frequency at 0.5, 1.0, 2.0, 4.0, 8.0, 16.0 Hz, (b) of temporal frequency at 1.5 Hz and spatial frequency at 0.01, 0.02, 0.04, 0.08, 0.16, 0.32 cpd, respectively, and their evoked maps of orientation selective pixels (pixel size: 7 μm; color-coded by preferred orientation) and maximal calcium responses across all orientations. Grating size: 38°. Representative result from 4 mice. (c, d) Averages of maximal responses for pixels within the dashed line at each (c) temporal and (d) spatial frequency for 4 mice. Upper panel: ΔF/F%; Lower panel: normalized to maximum of each trace in the upper panel; gray shade: s.d. Source data are provided as a Source Data file.

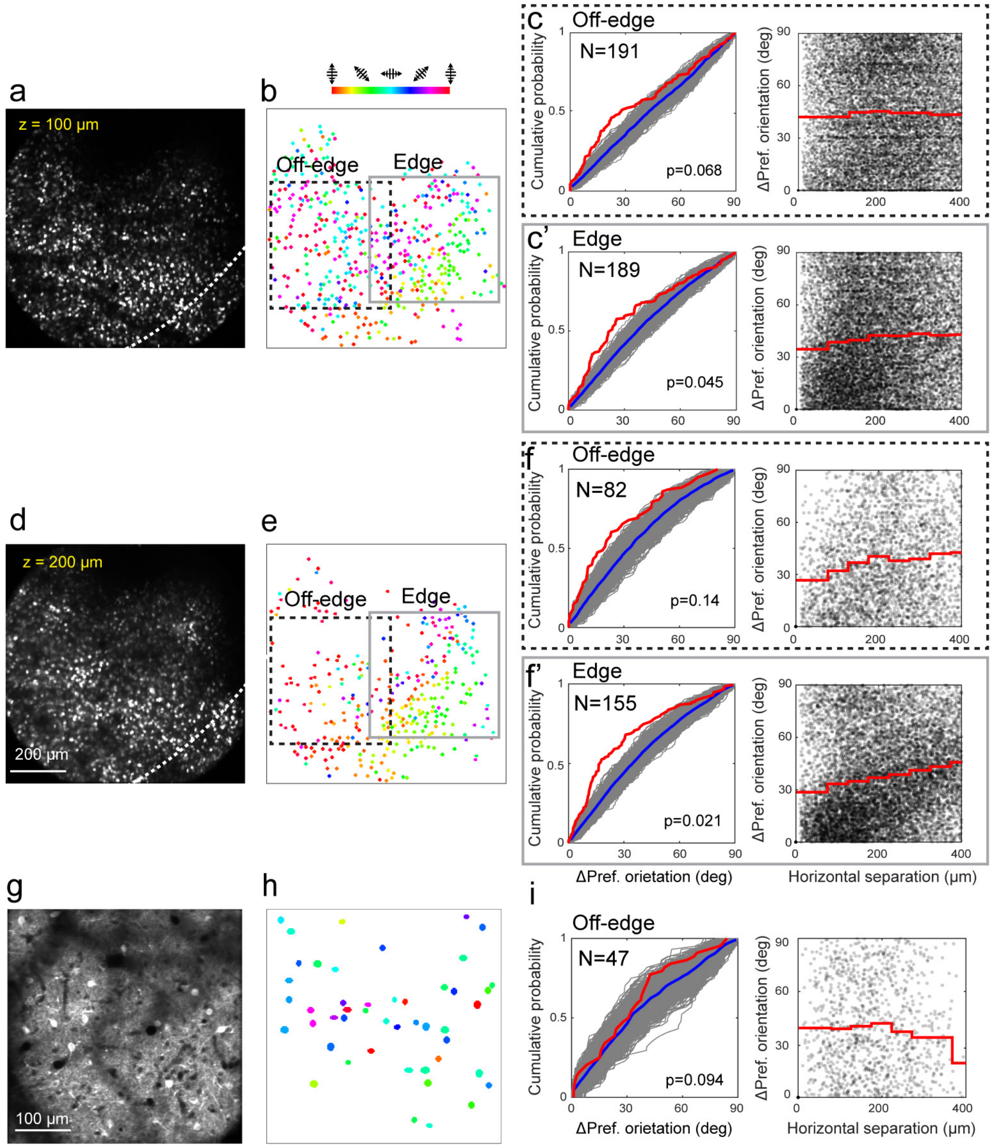

Supplementary Fig. 4. Lack of orientation clustering away from edge. (a,d) sSC neurons expressing nuclear-targeted GCaMP6s at different depths in a mouse with overlaying cortex removed. (b,e) OS neurons color-coded by their preferred orientation. Dashed line: approximate location of grating edge. (c,c',f,f') Left: Cumulative distributions of preferred orientation difference between nearest neighbors (red curve), randomly selected neuron pairs (1,000 repeats, gray curves), and the average of random distributions (blue curve); Right: Difference in preferred orientations for pairs of OS neurons versus their horizontal separation (red curve: averages of 50-µm bins), for neurons (c,f) away from or (c',f') near stimulus edge. p value: two-sample Kolmogorov-Smirnov test between red and blue curves. Representative result from N = 3 mice. (g,h,i) Similar analysis to (a,b,c) for neurons expressing GCaMP6s cytosolically. Representative result from N = 4 mice. Statistical tests were two-sided. a-e share the same scale bar.

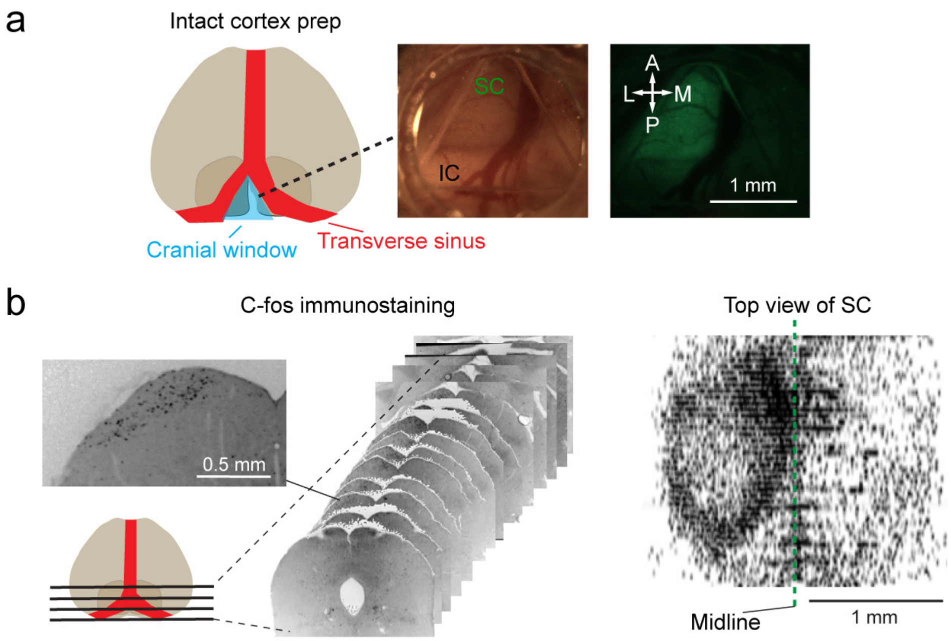

Supplementary Fig. 5 Cortex-intact preparation and C-fos experiment. (A) Left: A triangular glass plug pushed transverse sinus anteriorly to expose SC without cortex removal; Right: *In vivo* brightfield and widefield fluorescence images of medial posterior SC expressing nuclear-targeted GCaMP6s. (B) Left: Example brain sections immunostained for c-Fos from a mouse having been presented the circular grating stimuli in Figure 3 for two hours; Right: Dorsal view of the reconstructed volume from SC sections immunostained for c-Fos. Representative result from 4 mice.

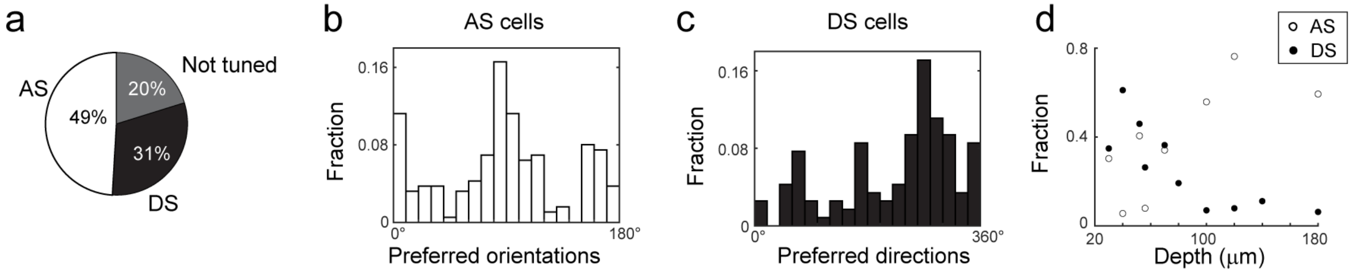

Supplementary Fig. 6. Orientation and direction selectivity of SC neurons towards full-field drifting gratings. (a). Fraction of axis-selective (AS), direction selective (DS) and non-orientation-tuned cells that showed visually evoked activity during full-field drifting grating stimuli. 380 neurons from 6 mice. (b) Distribution of preferred orientation of AS neurons (187 neurons, 6 mice). (c) Distribution of preferred direction of DS neurons (117 neurons, 6 mice). (d) Fraction of DS or AS cells as the function of depth. Each dot represents a FOV. 10 FOVs from 6 mice.

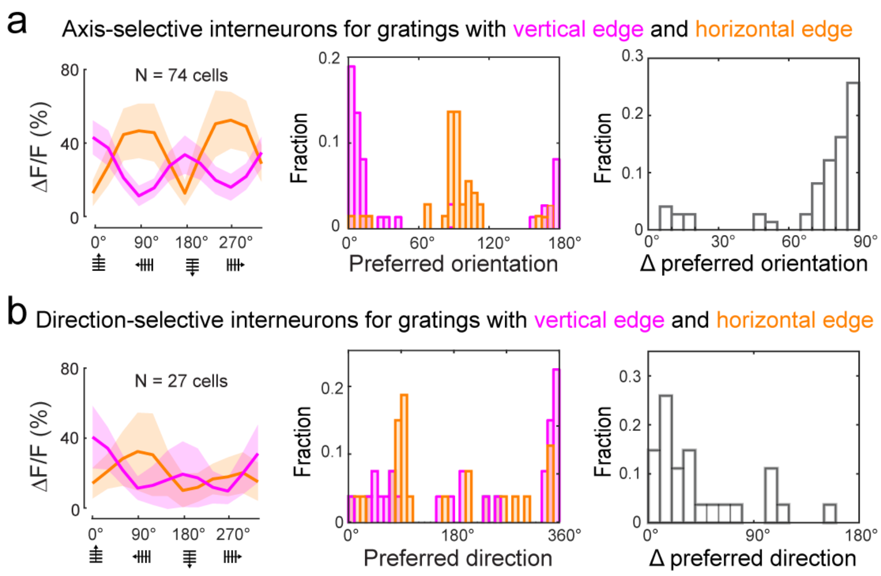

Supplementary Fig. 7. SC interneurons exhibit the same edge-induced orientation selectivity. (A) (From left to right) average calcium response ΔF/F%, preferred orientation of axis-selective neurons in response to gratings with vertical edge or horizontal edge, and change in preferred orientation for individual neurons. 74 neurons from 6 mice. (B) (From left to right) average calcium response ΔF/F%, preferred direction of direction-selective neurons in response to gratings with vertical edge or horizontal edge, and change in preferred direction for individual neurons. 27 neurons from 6 mice. Shaded error bands: s.d..

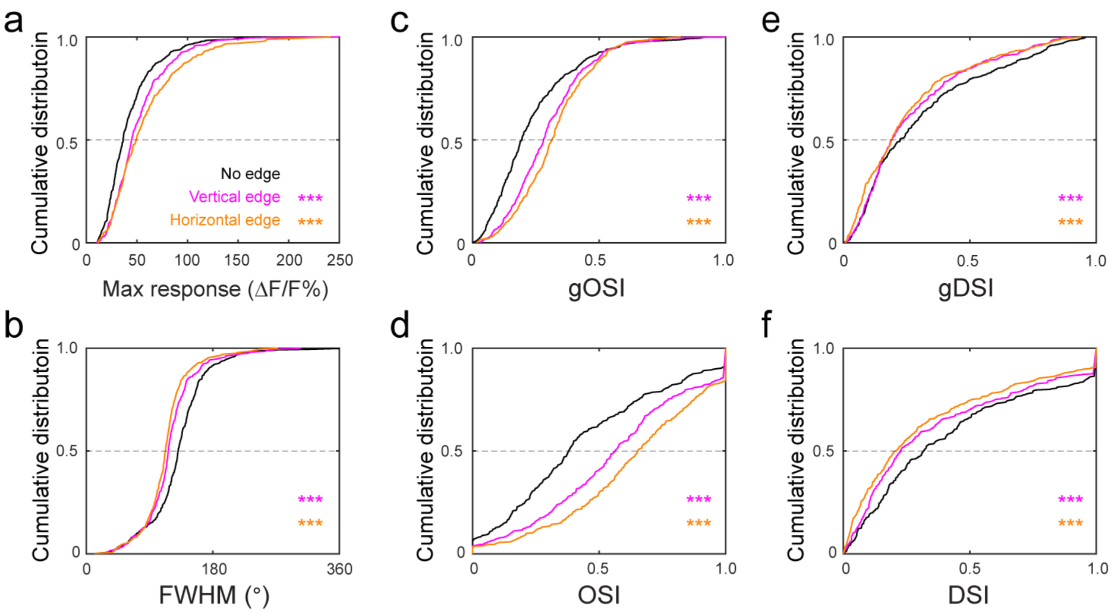

Supplementary Fig. 8 Response and tuning properties of SC neurons towards drifting grating stimuli without edge, with vertical edge, or with horizontal edge. Cumulative distributions of (a) max responses, (b) FWHM, (c) gOSI, (d) OSI, (e) gDSI, and (f) DSI for 363 cells from 6 mice. Two-sided Wilcoxon signed rank test was used to compare population properties towards drifting gratings without edge with the properties towards gratings with vertical or horizontal edge. *$p < 0.05$, **$p < 0.01$, ***$p < 0.001$. p values (vertical, horizontal): (a) (5.2×10^{-17}, 2.3×10^{-4}); (b) (6.6×10^{-4}, 3.6×10^{-6}); (c) (2.3×10^{-10}, 2.8×10^{-21}); (d) (8.2×10^{-10}, 1.8×10^{-24}); (e) (8.6×10^{-4}, 3.6×10^{-6}); (f) (9.1×10^{-5}, 3.9×10^{-9}).

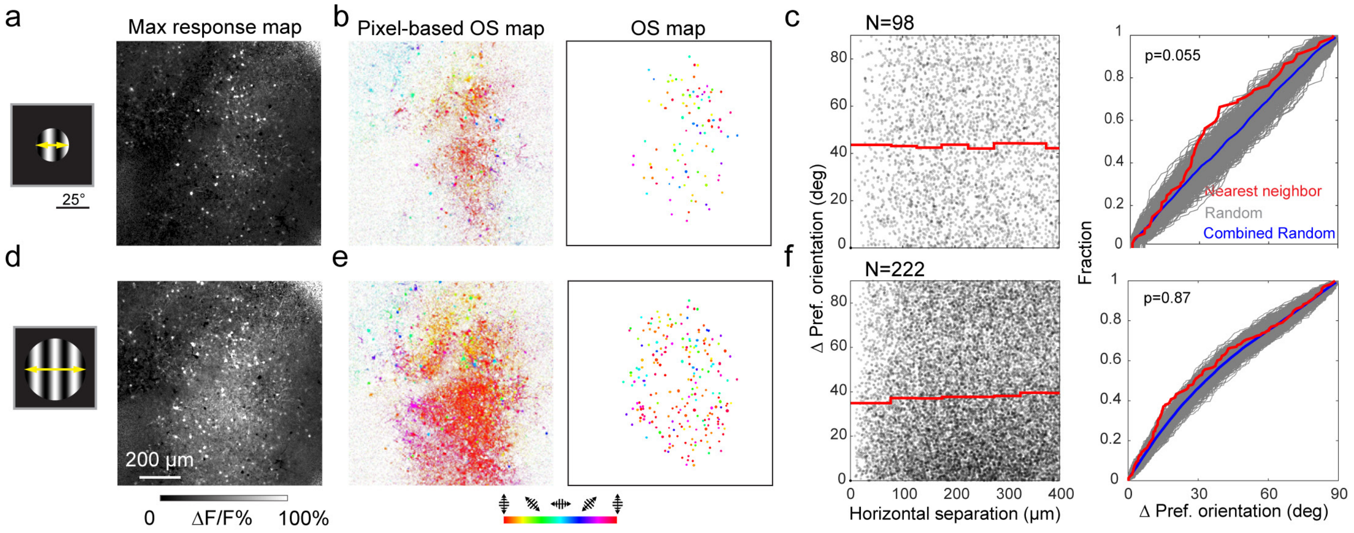

Supplementary Fig. 9. Mouse primary visual cortex lacks similar edge-induced effects. Primary visual cortical neurons labeled with AAV2/1.syn.GCaMP6s responding to circular drifting gratings of (a-c) 25° or (d-f) 50° view angles. (a,d) Maximal calcium responses across all orientations. (b,e) Maps of (left) OS pixels and (right) OS neurons, color-coded by their preferred orientation. (c,f) Left: Difference in preferred orientations for pairs of OS neurons versus their horizontal separation (red curve: averages of 50-µm bins). Right: Cumulative distributions of preferred orientation difference between nearest neighbors (red curve), randomly selected neuron pairs (1,000 repeats, gray curves), and the average of random distributions (blue curve). p value: two-sample Kolmogorov-Smirnov test between red and blue curves. Representative results reproduced in N=4 mice.

Supplementary Table 1. Imaging parameters associated with experimental data.

	Objective	Laser power (mW)	Depth (μm)	Pixel size (μm)
Figure 1c	4×, 0.28 NA	20	150	7.0
Figure 1d	4×, 0.28 NA	116	80	6.5
Supplementary Fig. 1	4×, 0.28 NA	48-116	50-100	6.0 or 7.0
Figure 2	4×, 0.28 NA	22	80	7.0
Supplementary Fig. 2	4×, 0.28 NA	22	80	7.0
Supplementary Fig. 3	4×, 0.28 NA	40 to 94	80-100	5.0-7.0
Figure 3	10×, 0.6 NA	29 to 109	50-150	3.0-3.8
Supplementary Fig. 4	10×,0.6 NA or 16×, 0.8 NA	51 to 89 or 13	100-200 or 52	3.2 or 1.5
Supplementary Fig. 6	16×, 0.8 NA	13-30	20-180	1.5
Figure 4	16×, 0.8 NA	13-30	20-180	1.5
Supplementary Fig. 7	16×, 0.8 NA	15-28	50-220	1.8
Figure 5	10×,0.6 NA or 16×, 0.8 NA	25-70	50-100	1.0-3.0
Supplementary Fig. 8	16×, 0.8 NA	15-28	50-220	1.8
Figure 6	4×, 0.28 NA	88	80	7.0
Supplementary Fig. 9	16×, 0.8 NA	30-100	150-300	1.0-1.5
