# Supplementary material for: Stimulus edges induce orientation tuning in superior colliculus
Source: Nat Commun. 2023 Aug 8;14:4756. doi: 10.1038/s41467-023-40444-1 (PMC10409754; doi:10.1038/s41467-023-40444-1)
Supplement: Supplementary file 3 — Reporting Summary [file 41467_2023_40444_MOESM3_ESM.pdf]

nature portfolio

Corresponding author(s): Na Ji

Last updated by author(s): Jun 20, 2023

Reporting Summary

Nature Portfolio wishes to improve the reproducibility of the work that we publish. This form provides structure for consistency and transparency in reporting. For further information on Nature Portfolio policies, see our Editorial Policies and the Editorial Policy Checklist.

Statistics

For all statistical analyses, confirm that the following items are present in the figure legend, table legend, main text, or Methods section.

n/a	Confirmed	
☐	☒	The exact sample size (*n*) for each experimental group/condition, given as a discrete number and unit of measurement
☐	☒	A statement on whether measurements were taken from distinct samples or whether the same sample was measured repeatedly
☐	☒	The statistical test(s) used AND whether they are one- or two-sided<br>*Only common tests should be described solely by name; describe more complex techniques in the Methods section.*
☐	☒	A description of all covariates tested
☐	☒	A description of any assumptions or corrections, such as tests of normality and adjustment for multiple comparisons
☐	☒	A full description of the statistical parameters including central tendency (e.g. means) or other basic estimates (e.g. regression coefficient) AND variation (e.g. standard deviation) or associated estimates of uncertainty (e.g. confidence intervals)
☐	☒	For null hypothesis testing, the test statistic (e.g. *F*, *t*, *r*) with confidence intervals, effect sizes, degrees of freedom and *P* value noted<br>*Give P values as exact values whenever suitable.*
☒	☐	For Bayesian analysis, information on the choice of priors and Markov chain Monte Carlo settings
☒	☐	For hierarchical and complex designs, identification of the appropriate level for tests and full reporting of outcomes
☒	☐	Estimates of effect sizes (e.g. Cohen's *d*, Pearson's *r*), indicating how they were calculated

Our web collection on statistics for biologists contains articles on many of the points above.

Software and code

Policy information about availability of computer code

Data collection	Custom-built 2-photon microscope controlled by custom code in LabVIEW 2015 (detailed in https://doi.org/10.1038/nmeth.1411) was used for data acquisition.
Data analysis	Custom-written codes in MATLAB (R2018a and R2021a) was used for image and data analysis can be found at https://github.com/JiLabUCBerkeley/SCimaging. Psychtoolbox versions 3.0.15 and 3.0.18 were used to generate visual stimulation.

For manuscripts utilizing custom algorithms or software that are central to the research but not yet described in published literature, software must be made available to editors and reviewers. We strongly encourage code deposition in a community repository (e.g. GitHub). See the Nature Portfolio guidelines for submitting code & software for further information.

Data

Policy information about availability of data

All manuscripts must include a data availability statement. This statement should provide the following information, where applicable:

- Accession codes, unique identifiers, or web links for publicly available datasets
- A description of any restrictions on data availability
- For clinical datasets or third party data, please ensure that the statement adheres to our policy

Source data are provided with this paper. All other data are available from the lead contact, Na Ji (jina@berkeley.edu), upon reasonable request.

Research involving human participants, their data, or biological material

Policy information about studies with human participants or human data. See also policy information about sex, gender (identity/presentation), and sexual orientation and race, ethnicity and racism.

Reporting on sex and gender	N/A
Reporting on race, ethnicity, or other socially relevant groupings	N/A
Population characteristics	N/A
Recruitment	N/A
Ethics oversight	N/A

Note that full information on the approval of the study protocol must also be provided in the manuscript.

Field-specific reporting

Please select the one below that is the best fit for your research. If you are not sure, read the appropriate sections before making your selection.

☒ Life sciences ☐ Behavioural & social sciences ☐ Ecological, evolutionary & environmental sciences

For a reference copy of the document with all sections, see nature.com/documents/nr-reporting-summary-flat.pdf

Life sciences study design

All studies must disclose on these points even when the disclosure is negative.

Sample size	Our sample sizes, in terms of the numbers of animals studied, optical sections imaged, and neurons analyzed, are similar to or larger than those generally employed when 2-photon fluorescence microscopy was used to image the mouse brain (e.g., https://doi.org/10.1038/nature14103)
Data exclusions	No data were excluded from the analyses.
Replication	We confirmed our findings as reproducible in 4 (for Fig. 5) or 6 (for all other figures) mice, with measurements done on each mouse independent of those carried out on other mice.
Randomization	Randomization on the level of samples does not apply to our study, because all the mice underwent the same surgery to introduce fluorescence indicators into their brains. Only brains with fluorescence indicator expression can be used for our imaging study.
Blinding	Because we can only use brains with fluorescence indicator expression for our in vivo imaging study and because we are not separating mice into different experimental groups, blinding is not relevant to our experimental design.

Reporting for specific materials, systems and methods

We require information from authors about some types of materials, experimental systems and methods used in many studies. Here, indicate whether each material, system or method listed is relevant to your study. If you are not sure if a list item applies to your research, read the appropriate section before selecting a response.

Materials & experimental systems

n/a	Involved in the study	
☐	☒	Antibodies
☒	☐	Eukaryotic cell lines
☒	☐	Palaeontology and archaeology
☐	☒	Animals and other organisms
☒	☐	Clinical data
☒	☐	Dual use research of concern
☒	☐	Plants

Methods

n/a	Involved in the study	
☒	☐	ChIP-seq
☒	☐	Flow cytometry
☒	☐	MRI-based neuroimaging

Antibodies

Antibodies used	anti-c fos, Cell Signaling, c-Fos (9F6) Rabbit mAb #2250<br>Goat anti-Rabbit IgG Alexa Fluor 594, ThermoFisher, # A-11012
Validation	Statements from manufacturer's website: https://www.cellsignal.com/products/primary-antibodies/c-fos-9f6-rabbit-mab/2250 and https://www.thermofisher.com/antibody/product/Goat-anti-Rabbit-IgG-H-L-Cross-Adsorbed-Secondary-Antibody-Polyclonal/A-11012

Animals and other research organisms

Policy information about studies involving animals; ARRIVE guidelines recommended for reporting animal research, and Sex and Gender in Research

Laboratory animals	Adult C57BL/6 and Gad2-IRES-cre (Jax no. 010802) mus musculus were used.
Wild animals	No wild animals were used in the study.
Reporting on sex	Mice of both sexes were used.
Field-collected samples	No field-collected samples were used in the study.
Ethics oversight	All experimental protocols were conducted according to the National Institutes of Health guidelines for animal research and were approved by the Institutional Animal Care and Use Committee at Janelia Research Campus, Howard Hughes Medical Institute and at the University of California, Berkeley.

Note that full information on the approval of the study protocol must also be provided in the manuscript.
